# Interpersonal Violence Against Indigenous Sámi and Non-Sámi Populations in Arctic Sweden and the Mediating Effect of Historical Losses and Discrimination

**DOI:** 10.1177/08862605241264544

**Published:** 2024-09-10

**Authors:** Johanna Simmons, Christina Storm Mienna, Maria Josefsson, Per Axelsson, Katarina Nägga

**Affiliations:** 1 Linköping University, Sweden; 2 Umeå University, Sweden

**Keywords:** violence exposure, child abuse, cultural contexts, domestic violence, intergenerational transmission of trauma, community violence, intimate partner violence

## Abstract

The prevalence of interpersonal violence has been reported at higher levels among Indigenous than non-Indigenous populations worldwide, but has not been thoroughly investigated among the Sámi population in Sweden. The aims of this study were to investigate: (1) the prevalence of emotional, physical, and sexual violence and violence by intimate partners, family members, acquaintances, and strangers among participants identifying as Sámi or Swedish, (2) whether reporting experiences of historical losses and discrimination mediated the anticipated association between identifying as Sámi and reporting experiences of violence, and (3) whether background characteristics were associated with reporting experiences of violence. Cross-sectional questionnaire data collected in 2021 for the “Health and Living conditions in Sápmi” study were used. All adults in an arctic region in Sweden were invited to participate (response rate: 41%). Respondents self-identifying as Sámi (*n* = 375; 24.7%) or Swedish (*n* = 1,144; 75.3%) were included in this study. Sámi respondents of both sexes more often reported violence by an acquaintance or stranger. Likewise, more Sámi than Swedish women reported family violence (16.4% vs. 9.2%), but there was no difference concerning intimate partner violence (13.3% vs. 15.4%). Mediation analyses revealed strong positive indirect effects of historical losses and discrimination on the different types of violence. Being female was the strongest predictor of reporting intimate partner violence, and younger age was associated with violence by all perpetrators except family members. In conclusion, interpersonal violence was more often reported by Sámi respondents, but the association was explained in full by experiences of historical losses and discrimination. The results underline the importance of a life-course and even intergenerational and historical perspectives when investigating interpersonal violence.

## Background

Studies have revealed higher prevalence rates of exposure to interpersonal violence among Indigenous than non-Indigenous populations worldwide, for example, childhood abuse and intimate partner violence in Canada (Brownridge, 2008; Brownridge et al., 2017; Kwan, 2015), intimate partner violence in the USA (Oetzel & Duran, 2004), and physical and sexual violence during the life-course in Australia (Nasir et al., 2021). However, the prevalence of interpersonal violence has only partly been investigated among the Indigenous Sámi population in the Nordic countries (Burman, 2017; Eriksen et al., 2015). A recent study reported the prevalence of sexual abuse regardless of perpetrator among Sámi men and women (Brandén et al., 2023), and more extensive data are available from the SAMINOR 2 study, conducted in Norway. In the latter, a higher prevalence of overall life-course victimization, as well as childhood abuse and intimate partner violence, was found among the Indigenous Sámi than non-Sámi population (Eriksen et al., 2015, 2021). In this study, the prevalence of interpersonal violence among respondents identifying as Sámi or Swedish in the municipality of Jokkmokk in Arctic Sweden was investigated. In addition, two factors that potentially explain the higher prevalence rate of interpersonal violence repeatedly found among Indigenous populations were explored: historical losses and experiences of discrimination.

**Figure 1.**
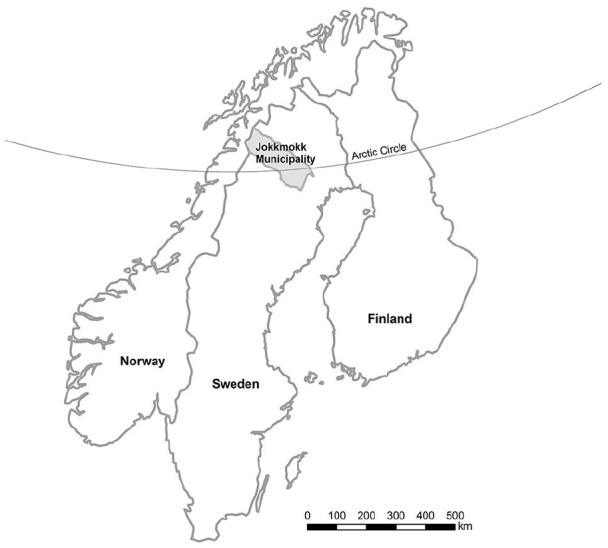
Map of Jokkmokk.

The Health and Living conditions in Sápmi (HALDI) study began its collaboration with Jokkmokk’s municipality in 2015. Jokkmokk is situated in the inlands of Arctic Sweden (Figure 1) and is part of Sápmi, that is, the traditional Sámi land that includes northern parts of Norway, Sweden, Finland, and Russia’s Kola Peninsula. Jokkmokk has been known for having a strong Sámi presence for centuries, and until the mid-1800s the Sámi population was a majority in the region (Skold et al., 2011). Historically, the Sámi population depended on reindeer husbandry, fishing, or agriculture, and many lived nomadic lives, moving across the Sápmi territory with their reindeer herds. Colonial practices on the part of the state and the church toward the Sámi population have a long history, dating back to the 17th century and culminating in severe assimilation policies during the 19th and 20th centuries. For the Sámi population in Jokkmokk and in Sweden this resulted in, for example, forced dislocation, forced participation in eugenics studies, and forced assimilative boarding schools for Sámi children. As a result, many Sámi people lost their livelihood, language, and culture, and many Sámi children were exposed to violence in boarding schools (Huuva & Blind, 2016). However, since the mid-1900s, a Sámi political movement has led to the establishment of Sámi organizations and a Sámi parliament (Lantto & Mörkenstam, 2008). Today, Jokkmokk municipality is the home of several Sámi cultural institutions and organizations, a famous winter market dating back to 1605, and since 2021, a resource center that specializes in Sámi health.

In the last two decades, studies on intergenerational trauma, historical trauma, and collective trauma in relation to Indigenous populations have emerged globally. This line of research focuses on collective, repetitive trauma inflicted over generations on a group of people that share a commonality, for example, ethnicity (Evans-Campbell, 2008; Whitbeck et al., 2004). A previous study found that 56% of Sámi respondents reported experiences of historical trauma (La Parra-Casado et al., 2023). There is no universal definition of historical trauma, but the most common definition assert that the legacy of colonization accrues across generation of Indigenous people, leading to shared vulnerabilities that undermine contemporary health status (Evans-Campbell, 2008; Gone et al., 2019). In this study, we consider different forms of historical losses, for example, loss of land, culture, and language, that are considered important parts of historical trauma. Although the term “historical” is used, historical losses are not confined to experiences of past generations; rather, it entail losses that are still present and affect the everyday lives of many Indigenous people (Whitbeck et al., 2004). In addition, experiences of contemporary injustices such as racism and discrimination are common among Indigenous peoples, and a recent study of the Sámi found that 41% of respondents reported experiences of discrimination because of being Sámi (Evans-Campbell, 2008; La Parra-Casado et al., 2023). The importance of considering both historical losses and contemporary injustices in studies of Indigenous populations has repeatedly been asserted, as has the interaction between the two (Bombay et al., 2014; Burnette, 2015; Kirmayer et al., 2014).

One theory that can be used to understand how direct interpersonal violent events are related to and legitimized by structural and cultural forms of violence is the “violence triangle” (Galtung, 1990). The theory is illustrated by putting direct, structural, and cultural violence as the corners in a triangle. Examples of structural violence are exploitation and marginalization while cultural violence is defined as any aspect of culture that can be used to legitimize direct or structural violence, for example, religion, ideology, and language (Galtung, 1990). Sámi people’s experiences of colonialism have been suggested to be considered an expression of structural and cultural violence (Sehlin MacNeil, 2018). This is in line with theories stating that colonial violence endured by Indigenous peoples worldwide may lead to an internalization of oppression that can be passed on through generations and affect thoughts, behaviors, and relationships, and lead to a normalization of violence as a strategy for solving conflict (Brownridge et al., 2017; Burnette & Hefflinger, 2017; Hoffart & Jones, 2018; Kwan, 2015). The relationship between cultural, structural, and direct forms of violence is likely to be an important factor in understanding why Indigenous people tend to report higher prevalence rates of interpersonal violence than non-Indigenous people. It is therefore important to investigating if experiences of historical losses and discrimination mediate the association often found between ethnicity and exposure to interpersonal violence for Indigenous people.

Another often-used theoretical framework for understanding interpersonal violence is the socioecological model, and it has also been used in Indigenous populations (Bronfenbrenner, 1986; Heise, 1998; Oetzel & Duran, 2004). The model shows that there are risk and protective factors for interpersonal violence on the individual, relational, community, and societal levels, and that factors on all levels intersect and affect the risk of violence. On the individual level of the ecological model, studies about poly-victimization have repeatedly established that one of the strongest risk factors for violence is previous exposure to violence (Hamby & Grych, 2013; Simmons et al., 2014). In addition to direct forms of violence, exposure to indirect forms of violence, for example, witnessing parental violence, has been found to have negative long-term effects for those exposed (Vu et al., 2016). Hamby et al. (2019) argue that direct colonial violence experienced by older generations of Indigenous populations can also be considered to be a form of indirect violence for younger generations as they are exposed by being told about or witnessing the negative effects of violence. Theories about historical losses and other forms of historical trauma therefore add to the poly-victimization framework by pointing out that violence can also be endured collectively, and that the burden of trauma can be transmitted through generations (Hamby et al., 2019). In addition, experiences of oppression (societal or community level) may result in what has been labeled “lateral violence” or “horizontal violence,” (relational level) meaning that violence directed at intimate partners, family members, and peers may be a consequence of historical oppression, as people who are oppressed tend to oppress others by, for example, lashing out violently toward those in their vicinity (Whyman et al., 2021). Altogether, colonialism can be considered an example of how the different levels of the socioecological model intersect and contribute to an increased risk of exposure to interpersonal violence. In addition, previous studies have revealed higher levels of several risk factors for interpersonal violence on different levels of the socioecological model among Indigenous than non-Indigenous populations. Low socioeconomic status, high unemployment rates, and high alcohol consumption, for example, are reported among Indigenous Peoples in Canada as compared to non-Indigenous Canadians, and this was found to partially account for increased odds of reporting intimate partner violence among the former group (Brownridge, 2003, 2008). The ecological model also suggests that risk factors for violence that are not specific to Indigenous populations, for example, gender and age, should be considered.

In conclusion, interpersonal violence has previously been reported at higher levels among Indigenous than non-Indigenous populations worldwide but has not been thoroughly investigated in Sweden. Reasons for the elevated prevalence among Indigenous populations may well include collective experiences of historical losses and discrimination, but this needs to be further explored while also considering other factors on different levels of the socioecological model. Therefore, the aims of this study were to investigate:

1.  The prevalence of emotional, physical, and sexual violence as well as any violence by intimate partners, family members, acquaintances, and strangers among participants identifying as Sámi or Swedish.
2.  Whether reporting experiences of historical losses and discrimination mediated the anticipated association between identifying as Sámi and reporting experiences of violence.
3.  Whether background characteristics on different levels on the socioecological model, including experiences of historical losses and discrimination, were associated with reporting experiences of interpersonal violence.

## Method

### Sample

Data collected in 2021 for the HALDI, Sweden study were used. The study was approved by the Swedish Ethical Review Authority (Dnr 2020-03662). During the HALDI study, all adults (aged 18 and above) in the municipality of Jokkmokk in Arctic Sweden were invited to participate by answering a questionnaire (*n* = 4,077) that was available in three languages: Lule-Sámi, North-Sámi, and Swedish. In total, 1,682 questionnaires (41%) were returned. To ensure Sámi involvement and that the questions and research methods used in the project were culturally appropriate and relevant, 11 focus-group discussions with a total of 51 Sámi informants were conducted before the study begun (Storm Mienna et al., 2021). One of the important findings of the focus groups was that measures of ethnicity should be based on self-identification, and therefore, one question was used for this purpose: *How do you identify yourself?* The possible answers were (a) Swedish, (b) Sámi, (c) Other, please specify. It was possible to choose more than one category. Only those classified as “Sámi” (*n* = 375) or “Swedish” (*n* = 1,144) were included in the current study (*n* = 1,519). A respondent identifying as either only “Sámi,” or as “Sámi” and “Swedish,” or “Sami” and “Other,” was classified as “Sámi.” Similarly, those identifying as “Other” or “Other” and “Swedish” were classified as “Other,” and hence excluded from the current study.

### Measurement

In total, 112 questions covering topics relating to the respondents’ health and living conditions were included. One question was used for each specific form of violence. Physical: *Have you been subjected to some form of physical violence (e.g., being pushed, grabbed, hit, or kicked, or having things thrown at you)?* Emotional: *Have you experienced someone systematically and over a long period of time trying to control, repress, degrade, or humiliate you?* Sexual: *Have you been subjected to some form sexual violence (e.g., someone touching you against your will or subjecting you to sexual actions)?* The response categories for the questions were: (a) Yes as a child, (b) Yes as an adult, (c) No. After each violence-specific question, a follow-up question was asked about the perpetrator: (a) A stranger, (b) A family member/relative (labeled “Family”), (c) A husband/wife/partner (labeled “Partner”), or (d) Other known persons (labeled “Acquaintances”). It was possible to choose more than one category both concerning when the violence occurred and the kind of perpetrator. Respondents who did not answer any of the violence specific questions (*n* = 16) were coded as missing on all items about exposure to violence. When constructing the variables about kind of perpetrator and repeat victimization, respondents with missing values on one or two of the questions about type of violence were coded as nonexposed to that type of violence. This approach was taken to enable inclusion of respondents in the variables constructed to include multiple types of violence, without overestimating the prevalence of violence. A detailed description of the item nonresponse for the violence specific questions is included as part of the Supplemental Appendix.

The following question was used as a screening question for historical losses: *Do you have negative memories, or have you had negative experiences, concerning yourself or someone in your family, that currently affect you*? Respondents who answered “Yes” were asked: *How often do you think about the following: (a) Loss of land, (b) Loss of language, (c) Loss of family connections due to*, for example, *boarding school, fleeing from your homeland, or forced dislocation, (d) Loss of culture/lifestyle, (e) Loss of religion, (f) Other, please specify.* The response categories were “Every day,” “Every week,” “Every month,” “Every year or on special occasions,” and “Never.” Those that reported ever thinking about losses in relation to items (a) to (e) were classified as having experiences of historical losses. Those who reported only thinking about “Other” losses (f) were excluded because some of the examples given in these categories were events covered by other items in this study, for example, “intimate partner violence,” or negative experiences not necessarily related to historical losses, for example, “suicide by a family member” or “divorce.” The importance of including questions about experiences that can be labeled historical losses was put forward in the focus-groups preceding the current study (Storm Mienna et al., 2021). At the time of data collection, such questions had not been present in a population-based survey in Sweden or in Sápmi and the questions used were modified using the historical loss scale (Whitbeck et al., 2004). Due to limitations concerning the number of items in the questionnaire, the historical scale was shortened and has not been validated in the form used for this study.

The topic of discrimination was introduced by stating that discriminations occurs when a person or group of people is treated less favorably than others due to, for example, sex, transgender identity or expression, ethnicity, religion or other beliefs, disability, sexual orientation, or age. The respondents were then asked whether they had experienced discrimination (a) in the last 2 years and (b) prior to the last 2 years. The possible answers were “Never or very rarely,” “Rarely,” “Sometimes,” “Quite often,” and “Very often or always.” The answers to the two questions were merged into one item about ever experiencing any form of discrimination.

One question was used to estimate economic margin: *Would you or your household be able to pay an unexpected fee of 12,000 Swedish kronor within a month, without borrowing or asking for help?* Possible answers were “yes,” “no,” and “don’t know” One question was used as a proxy for social support: *Do you have someone that you can confide in? Possible answers were* “Yes, always,” “yes, most of the time,” “no, mostly not,” “no, never.” The questions about economic margin and social support were modified from similar questions used in the Swedish national public health survey (Public Health Agency of Sweden, 2020). Another question was used to measure alcohol consumption: *How often have you drunk alcohol in the last year?* That question was borrowed from the The Alcohol Use Disorders Identification Test (AUDIT-C) screening tool (Reinert & Allen, 2002). The respondents were also asked if they identified as “Male,” “Female,” “Other,” or “Prefer not to say.” Two participants responded “Other,” and four preferred not to say. Due to the low number of respondents in these categories, they were coded as “missing cases” concerning gender in all multivariate analysis. Age was derived from the question “What year where you born?”. Educational level was measured using the following item “What is your highest completed level of education?” Because the names of different educational levels in the Swedish school system have changed over the years, categories were made based on the number of years of educations (response categories in parentheses), ≤9 years (“elementary school, primary school, or similar”); 10 to 13 years (“2 years of upper secondary school or high school,” “3–4 years of upper secondary school or high school,” “residential college for adult education”) Higher education (“University or college, less than 3 years,” “University or college, 3 years or more”) Other (“Other education”).

### Statistical Analyses

Descriptive analysis concerning background characteristics (Table 1) was calculated using SPSS Statistics (version 29; IBM Corp., 2023). All other analyses were carried out using R Statistical Software (version 4.2.1; R Core Team, 2022) and using specific packages as described further on for each analysis.

**Table 1.** Background Characteristics of Total Sample (*n* = 1,519).

	Swedish		Sámi		*p*
Background Characteristics	*n*	%	*n*	%	
Total	1,144	75.3	375	24.7	
Gender					
Male	505	44.5	123	33.0	**<.01**
Female	629	55.4	246	66.0	
Other	0		2		
Prefer not to say	1		2		
Item nonresponse	9	0.8	2	0.5	
Age group (years)					
18–34	96	8.4	76	20.3	**<.01**
35–49	133	11.6	90	24.0	
50–69	469	41.0	139	37.1	
70 and older	446	39.0	70	18.7	
Item nonresponse	—		—		
Education					
≤9 years	287	25.2	47	12.6	**<.01**
10–13 years	463	40.7	140	37.5	
Higher education	334	29.3	174	46.6	
Other	54	4.7	12	3.2	
Item nonresponse	6	0.5	2	0.5	
Economic margin					
Yes	915	82.8	285	76.6	**<.01**
No, don’t know	190	17.2	87	23.4	
Item nonresponse	39	3.4	3	0.8	
Alcohol consumption					
Never	220	19.5	73	19.7	.09
≤1 times per month	347	30.8	138	37.2	
2–4 times per month	430	38.2	127	34.2	
2 or more times per week	130	11.5	33	8.9	
Item nonresponse	17	1.5	4	1.1	
Someone to confide in					
Always	671	60.1	215	58.0	.29
Most of the time	333	29.8	125	33.7	
Rarely or never	113	10.1	31	8.4	
Item nonresponse	27	2.4	4	1.1	
Historical losses					
No	1,018	96.5	210	59.8	**<.01**
Yes	37	3.5	141	40.2	
Item nonresponse	89	7.8	24	6.4	
Discrimination					
No, rarely	998	89.3	226	60.6	**<.01**
Sometimes	75	6.7	97	26.0	
Often, always	44	3.9	50	13.4	
Item nonresponse	27	2.4	2	0.5	

*Note.* Numbers in bold are significant differences between respondents identifying as “Swedish” or “Sámi” at the *p* = .05 level, calculated using Pearson’s chi-square test for each item. For the gender item, the chi-square test only included those respondents identifying as male or female, because the categories “other” and “prefer not to say” were small.

Pearson’s chi-square test were used to test for differences in background characteristics Because considerable differences were found between respondents identifying as “Swedish” and “Sámi” concerning age and gender, post-stratification weights balanced for age and gender were applied to the prevalence rates. There are no available data on ethnicity in Swedish population registers; therefore, the weights were based on overall population rates in Jokkmokk, 2021 obtained from Statistics Sweden. The weights were computed as the inverse of the probability of inclusion to the sample for each gender by age-group, where age was first stratified into approximately 5 years intervals (i.e., 18–25, 26–30, 31–35, . . ., 86–90, 91–). Weighted Pearson’s chi-square tests were used to test for differences in proportions of reported violence between those identifying as “Swedish” and “Sámi” (Aim 1). Analyses concerning prevalence rates were stratified by gender because men and women tend to report violence by different types of perpetrators, for example, women have repeatedly been found to report violence by an intimate partner more often than men when different forms of interpersonal violence are included in the same study (Hamby, 2016).

A bivariate mediation analysis using generalized linear regression (Yu & Li 2017) was used to investigate whether historical losses and discrimination mediated the effect of identifying as Swedish or Sámi on reporting interpersonal violence (Aim 2). The model is illustrated in Table 3. The analyses were performed in two steps. In the first step, for each of the interpersonal violence variables, it was tested whether historical losses and discrimination were identified as mediators. For a variable to be identified as a mediator, it must satisfy two conditions; (a) the variable is significantly correlated with the predictor (i.e., identifying as Swedish or Sámi), and (b) the variable is significantly correlated with the outcome (i.e., interpersonal violence), when adjusting for the confounding factors age, gender, education, economic margin, someone to confide in, and alcohol consumption in the model. Historical losses and discrimination were both identified as mediators for all of the interpersonal violence variables. Hence, in a second step, we performed multivariable bivariate mediation models for each interpersonal violence outcome variable fitted using logistic regression models, that is, a covariate adjusted mediation model with two mediators and a binary interpersonal violence outcome variable. The mediation effects and confidence intervals were estimated based on the estimated mediation effects from bootstrap samples. Model fit was assessed by monitoring the convergence of the bootstrap samples until sufficiently small Monte Carlo errors were obtained (*N* = 1,000). For all models, the results are presented as logodds of experiencing interpersonal violence. The function *data.org* was used to identify mediators and the mediation analyses were performed using the *boot.med* function in the R-package *mma* (Yu & Li, 2017). Binary logistic regression models were used to investigate the associations between background factors and odds of reporting interpersonal violence (Aim 3). One model was constructed for any life-course victimization as well as for life-course victimization by each kind of perpetrator, that is, violence by an intimate partner, a family member, acquaintance, or stranger. The study was not sufficiently powered to perform gender stratified analyses for Aims 2 and 3, but all the models were adjusted for background factors, that is, gender, age, education, economical margin, social support, alcohol consumption). There was a considerable nonresponse on especially the items regarding historical losses (respondents identifying as Swedish *n* = 89, 7.8%, respondents identifying as Sámi *n* = 24, 6.4%) (Table 1). For the mediation analyses as well as for the logistic regression analyses, an imputation procedure was therefore used to replace missing responses on single items using an expectation–maximization with bootstrapping method, as implemented in the Amelia II software package (Table 1) (Honaker et al., 2011).

## Results

Background characteristics of the sample can be found in Table 1. Altogether, 75.3% (*n* = 1,144) of the sample identified as Swedish and 24.7% (*n* = 375) as Sámi. Sámi respondents were more likely to be female, while the Swedish respondents were older. Sámi respondents were more likely to report higher education (college or university) compared to the Swedish respondents. A larger proportion of those identifying as Sámi compared to Swedish reported having no economical margin, but there was no difference between the groups considering social support or alcohol consumption (Table 1).

### Aim 1

The weighted prevalence rates for men and women are presented in Table 2. To enable comparisons, the weighted and unweighted prevalence rates are presented together in the Supplemental Appendix. Only a small number of men reported sexual violence (Sámi *n* = 6, Swedish *n* = 13) as well as violence by a partner (Sámi *n* = 1 Swedish *n* = 11) or family perpetrator (Sámi *n* = 7, Swedish *n* = 30), and prevalence rates for these categories are therefore not presented. The chi-square test revealed that the respondents identifying as Sámi (men: *n* = 59, 47.7%, women: *n* = 126, 49.7%) reported life-course experiences of any violence more often than those identifying as Swedish (men: *n* = 165, 33.5%, women: *n* = 224, 37.0%). The same pattern was seen for both sexes concerning physical and emotional violence and for women concerning sexual violence. Due to a low number of male respondents reporting sexual violence, differences between male respondents identifying as Swedish or Sámi could not be calculated.

**Table 2.** The Weighted Prevalence of Violence Exposure Among Men (*n* = 628) and Women (*n* = 875).

	Men					Women				
Kind of violent exposure	Swedish		Sámi			Swedish		Sámi		
	*n*	%	*n*	%	*p*	*n*	%	*n*	%	*p*
Any violence exposure					**<.01**					**<.01**
Yes	165	33.5	59	47.7		224	37.0	126	49.7	
No	333	66.5	64	52.3		397	63.0	119	50.3	
Item nonresponse	7		0			8		1		
Age at victimization					**<.01**					**<.01**
Only childhood	96	20.1	29	24.5		73	12.2	29	11.9	
Only adulthood	48	9.4	13	10.2		98	16.2	51	19.5	
Both childhood and adulthood	21	4.0	17	13.0		53	8.7	46	18.4	
Type of violence										
Emotional	72	14.4	29	24.7	**<.01**	125	21.3	71	28.8	**.02**
Physical	134	27.4	56	44.7	**<.01**	139	23.5	81	32.8	**<.01**
Sexual	N/A					115	19.1	80	31.7	**<.01**
Number of types					**<.01**					**<.01**
One type	113	23.3	29	23.3		112	18.3	52	20.3	
Two or more	52	10.2	30	24.4		112	18.7	74	29.4	
Kind of perpetrator										
Partner	N/A					93	15.4	35	13.3	.44
Family	N/A					54	9.2	40	16.4	**<.01**
Acquaintance	71	14.8	30	25.0	**<.01**	88	14.7	62	24.3	**<.01**
Stranger	72	14.3	32	26.3	**<.01**	53	8.8	39	15.8	**<.01**
Number of perpetrators					**<.01**					**<.01**
One perpetrator	146	29.9	46	37.6		162	26.7	83	32.4	
Two or more perpetrators	18	3.5	12	9.6		59	10.0	43	17.3	

*Note.* The differences between groups (Swedish and Sami) were calculated using weighted Pearson’s chi-square test. Boldface numbers signal differences at the *p* = .05 level. The weights, that is, the inverse of the probability of inclusion to the sample, adjusted for age and gender, were based on population rates in Jokkmokk, 2021 obtained from Statistics Sweden. N/A = not applicable (Prevalence not reported due to low number of participants in category).

Intimate partner violence was reported at the same rate among female respondents identifying as Sámi (*n* = 35, 13.3%) and respondents identifying as Swedish (*n* = 93, 15.4%; *p* = .44). Family violence was reported by 16.4% (*n* = 40) of women identifying as Sámi and 9.2% (*n* = 54) of women identifying as Swedish (*p* = <.01). Due to a low number of male respondents reporting intimate partner violence or family violence, differences between male respondents identifying as Swedish or Sámi could not be calculated. For both genders, respondents identifying as Sámi reported higher levels of violence perpetrated by an acquaintance (Sámi male: *n* = 30, 25.0%, Swedish: male *n* = 71, 14.8%; Sámi female: *n* = 62, 24.3%, Swedish female: *n* = 88, 14.7%) as well as by a stranger (Sámi male: *n* = 32, 26.3%, Swedish male: *n* = 72, 14.3%; Sámi female: *n* = 39, 15.8%, Swedish female: *n* = 53, 8.8%) (Table 2). Sámi respondents were more likely to report experiences of multiple victimization, that is, two or more perpetrators (Sámi male:12, 9.6%, Swedish male: *n* = 18, 3.5%; Sámi female: *n* = 43, 17.3%, Swedish female: *n* = 59, 10.0%), two or more forms of violence (Sámi male: *n* = 30, 24.4%, Swedish male: *n* = 52, 10.2%; Sámi female: *n* = 74, 29.4%, Swedish female: *n* = 112, 18.7%), and experiences of violence as both a child and adult (Sámi male: *n* = 17, 13.0%, Swedish male: *n* = 21, 4.0%; Sámi female: *n* = 46, 18.4%, Swedish female: *n* = 53, 8.7%) (Table 2).

### Aim 2

In the mediation analyses, identifying as Sámi had a significant total effect on reporting all forms of interpersonal violence (Table 3). The total effect on reporting intimate partner violence was negative, that is, respondents identifying as Sámi were less likely to report violence than those identifying as Swedish (*p* = .04). For any life-course victimization (*p* = .01) as well as violence by family members (*p* = .02), acquaintances (*p* = .02), and strangers (*p* = .02), the direct effect was positive, that is, respondents identifying as Sámi were more likely to report violence than those identifying as Swedish. The indirect effect of reporting discrimination was significant for all forms of violence, that is, identifying as Sámi was positively associated with reporting discrimination, and discrimination was in turn positively associated with reporting experiences of interpersonal violence. Except for violence by a stranger, the same was true for historical losses. The indirect effect of historical losses and discrimination completely explained the total effect of identifying as Sámi on interpersonal violence for any life-course victimization, violence by family members, acquaintances, and strangers. This is to say that identifying as Sámi had no direct effect on the likelihood of reporting these forms of interpersonal violence. For intimate partner violence, the direct effect of identifying as Sámi was negative, that is, respondents identifying as Sámi were less likely to report intimate partner violence.

**Table 3.** Effects of Identifying as Sámi, Reporting Experiences of Discrimination, and Reporting Historical Losses on Violence Exposure as Found in the Mediation Analyses, Adjusted for Covariates.

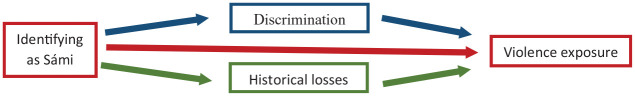			
Type of violence and kind of effect	Mediation effect	95% CI	*p*
Any violence			
Total effect	0.41	[0.10, 0.72]	.01
Direct effect (red)	−0.28	[−0.61, 0.03]	.08
Indirect effect discrimination (blue)	0.31	[0.20, 0.42]	<.01
Indirect effect historical losses (green)	0.36	[0.25, 0.54]	<.01
Family perpetrator			
Total effect	0.50	[0.09, 0.95]	.02
Direct effect (red)	−0.08	[−0.57, 0.35]	.63
Indirect effect discrimination (blue)	0.18	[0.07, 0.33]	<.01
Indirect effect historical losses (green)	0.42	[0.26, 0.61]	<.01
Partner perpetrator			
Total effect	−0.42	[−0.90, −0.03]	.04
Direct effect (red)	−0.80	[−1.31, −0.34]	.00
Indirect effect discrimination (blue)	0.14	[0.01, 0.28]	.04
Indirect effect historical losses (green)	0.24	[0.05, 0.40]	.01
Stranger perpetrator			
Total effect	0.47	[0.07, 0.82]	.02
Direct effect (red)	0.08	[−0.37, 0.48]	.81
Indirect effect discrimination (blue)	0.29	[0.17, 0.42]	<.01
Indirect effect historical losses (green)	0.10	[−0.06, 0.26]	.21
Acquaintance perpetrator			
Total effect	0.37	[0.08, 0.76]	.02
Direct effect (red)	−0.13	[−0.48, 0.27]	.59
Indirect effect discrimination (blue)	0.18	[0.08, 0.30]	<.01
Indirect effect historical losses (green)	0.33	[0.18, 0.49]	<.01

*Note.* Identifying as “Swedish” was used as a reference category in the analyses. Hence, the data presented in the table are for respondents identifying as “Sámi ”compared to those identifying as “Swedish,” for example, there was a positive association (total effect) between identifying as Sámi and reporting any life-course experiences of violence. This effect was explained in full by the indirect effect of discrimination (blue) and historical losses (green), resulting in a nonsignificant direct effect (red) of ethnicity on reporting any violence. The following variables were included in the analyses as covariates: gender, age, education, economical margin, social support and alcohol consumption. All respondents (*n* = 1,519) were included in the analyses and an imputation procedure was used to replace missing responses on single items using an expectation-maximization with bootstrapping method.

### Aim 3

Associations between background characteristics and reporting of different forms of interpersonal violence are presented in Table 4. The background characteristic most consistently associated with reporting experiences of violence was reporting experiencing discrimination often or always, for any victimization: *OR* = 4.98, 95% CI [2.90, 8.56]; family perpetrator: *OR* = 2.61, 95% CI [1.41, 4.83]; partner perpetrator: *OR* = 2.23, 95% CI [1.16, 4.29]; acquaintance perpetrator: *OR* = 2.44, 95% CI [1.49, 4.00]; stranger perpetrator *OR* = 3.88, 95% CI [2.33, 6.47]. Reporting historical losses was associated with: any victimization: *OR* = 3.10, 95% CI [2.12, 4.54], family violence: *OR* = 3.75, 95% CI [2.30, 6.11], intimate partner violence: *OR* = 1.75, 95% CI[1.02, 3.02], and violence by an acquaintance: *OR* = 2.69, 95% CI [1.80, 4.02], but not with reporting violence by a stranger: *OR* = 1.34, 95% CI [0.84, 2.14]. Being female was associated with higher odds of reporting intimate partner violence: *OR* = 7.95, 95% CI [4.34, 14.56], family violence: *OR* = 1.73, 95% CI [1.14, 2.63], as well as with lower odds for reporting violence by a stranger: *OR* = 0.40, 95% CI [0.29, 0.57]. Age ≥70 years was associated with lower levels of violence by all perpetrators except family members. Not having any economic margin was associated with intimate partner violence: *OR* = 1.86, 95% CI [1.21, 2.85] and reporting any victimization: *OR* = 1.39, 95% CI [1.02, 1.88]. Higher education was associated with reporting any victimization: *OR* = 1.70, 95% CI [1.19, 2.44] and violence by a stranger: *OR* = 1.82, 95% CI [1.06, 3.12], while “other” education was associated with reporting intimate partner violence: *OR* = 2.72, 95% CI [1.21, 6.09].

**Table 4.** Background Characteristics Associated with Reporting Interpersonal Violence.

Background characteristic	Any Victimization			Family			Partner			Acquaintance			Stranger		
	*OR*	95% CI		*OR*	95% CI		*OR*	95% CI		*OR*	95% CI		*OR*	95% CI	
Identifying as															
Swedish	1			1			1			1			1		
Sámi	**0.72**	**[0.53, 0.99]**		0.83	[0.51, 1.35]		**0.45**	**[0.27, 0.76]**		0.88	[0.61, 1.28]		1.00	[0.67, 1.49]	
Gender															
Male	1			1			1			1			1		
Female	0.97	[0.76, 1.24]		**1.73**	**[1.14, 2.63]**		**7.95**	**[4.34, 14.56]**		0.83	[0.61, 1.12]		**0.40**	**[0.29, 0.57]**	
Age															
18–34	1			1			1			1			1		
35–49	0.81	[0.52, 1.26]		1.23	[0.60, 2.50]		1.31	[0.72, 2.41]		0.68	[0.41, 1.10]		0.75	[0.45, 1.25]	
50–69	**0.59**	**[0.40, 0.86]**		1.71	[0.92, 3.20]		0.89	[0.51, 1.55]		0.70	[0.46, 1.07]		**0.50**	**[0.32, 0.80]**	
≥70	**0.33**	**[0.22, 0.50]**		1.46	[0.73, 2.91]		**0.41**	**[0.21, 0.80]**		**0.37**	**[0.22, 0.62]**		**0.30**	**[0.17, 0.52]**	
Education (years)															
≤9	1			1			1			1			1		
10–13	**1.70**	**[1.21, 2.39]**		1.30	[0.75, 2.25]		0.82	[0.46, 1.46]		1.40	[0.90, 2.18]		1.58	[0.94, 2.64]	
Higher education	**1.70**	**[1.19, 2.44]**		1.12	[0.64, 1.99]		0.89	[0.50, 1.59]		1.51	[0.95, 2.39]		**1.82**	**[1.06, 3.12]**	
Other	1.57	[0.85, 2.90]		0.64	[0.21, 2.01]		**2.72**	**[1.21, 6.09]**		0.99	[0.43, 2.30]		1.16	[0.46, 2.97]	
Economic margin															
Yes	1			1			1			1			1		
No	**1.39**	**[1.02, 1.88]**		0.99	[0.61, 1.60]		**1.86**	**[1.21, 2.85]**		1.35	[0.94, 1.94]		1.28	[0.85, 1.92]	
Someone to confide in															
Always	1			1			1			1			1		
Most of the time	**1.31**	**[1.01, 1.68]**		1.26	[0.84, 1.88]		0.96	[0.63, 1.46]		**1.39**	**[1.02, 1.90]**		1.09	[0.77, 1.55]	
Rarely or never	1.14	[0.76, 1.72]		0.91	[0.48, 1.73]		1.15	[0.61, 2.13]		1.28	[0.80, 2.04]		0.89	[0.52, 1.53]	
Alcohol consumption															
Never	1			1			1			1			1		
≤1 per month	0.94	[0.67, 1.32]		0.77	[0.46, 1.29]		1.11	[0.66, 1.87]		1.00	[0.66, 1.54]		1.10	[0.69, 1.75]	
2–4 times per month	0.99	[0.70, 1.38]		0.73	[0.43, 1.24]		1.21	[0.70, 2.07]		1.06	[0.69, 1.62]		0.91	[0.56, 1.47]	
≥2 per times week	1.17	[0.75, 1.82]		0.88	[0.43, 1.78]		1.07	[0.50, 2.30]		1.13	[0.65, 1.96]		0.99	[0.54, 1.82]	
Historical losses															
No	1			1			1			1			1		
Yes	**3.10**	**[2.12, 4.54]**		**3.75**	**[2.30, 6.11]**		**1.75**	**[1.02, 3.02]**		**2.69**	**[1.80, 4.02]**		1.34	[0.84, 2.14]	
Discrimination															
No. rarely	1			1			1			1			1		
Sometimes	**2.65**	**[1.84, 3.83]**		**1.94**	**[1.16, 3.25]**		**1.67**	[0.99, 2.80]		**1.81**	**[1.21, 2.72]**		**2.52**	**[1.63, 3.91]**	
Often. always	**4.98**	**[2.90, 8.56]**		**2.61**	**[1.41, 4.83]**		**2.23**	**[1.16, 4.29]**		**2.44**	**[1.49, 4.00]**		**3.88**	**[2.33, 6.47**	

*Note.* Boldface numbers signal differences at the *p* = .05 level. All variables presented in the table were included in analyses. All respondents (*n* = 1,519) were included in the analyses and an imputation procedure was used to replace missing responses on single items using an expectation-maximization with bootstrapping method. OR = odds ratios; CI = confidence interval.

## Discussion

We found a higher overall life-course prevalence of all considered types of interpersonal violence (physical, emotional, and for women sexual) among respondents identifying as Sámi than those identifying as Swedish. Likewise, both male and female Sámi respondents reported a higher prevalence of violence by acquaintances and strangers, and Sámi women also reported a higher prevalence of violence by family members. We did not, however, find any differences between women identifying as Swedish and Sámi concerning the reported prevalence of intimate partner violence. This is inconsistent with the results reported in previous studies of Indigenous populations in Canada and the USA, as well as the Norwegian SAMINOR 2 study (Brownridge et al., 2017; Eriksen et al., 2021; Oetzel & Duran, 2004). The prevalence of interpersonal violence is highly dependent on the survey instrument and methodology used (Hamby, 2016; Simmons & Swahnberg, 2019). Hence, comparisons of prevalence rates between samples are primarily meaningful when the same or a similar methodology and questions are used, making it difficult to compare these results with those of the studies conducted in Canada and in the USA, which used different instruments and methodologies. However, both in this study and the SAMINOR 2 study, items relating to violence were in part derived from the NorVold Abuse Questionnaire (NorAQ) (Swahnberg & Wijma, 2003). The prevalence of reported intimate partner violence among female respondents identifying as Swedish in the current study (15%) is similar to that reported in a previous study that used the NorAQ to study interpersonal violence toward women in Sweden (16%) (Simmons & Swahnberg, 2019), but higher compared to the non-Sámi female respondents in the SAMINOR 2 (12%). The prevalence of intimate partner violence among respondents identifying as Sámi (13%) was however lower in this study compared to Sámi women (17%) in the SAMINOR 2 (Eriksen et al., 2021).

Physical violence by any perpetrator was reported at considerably higher rates among both male and female respondents identifying as both Swedish and Sámi in the current study than in the SAMINOR 2 study. For example, in the current study, 27% of male respondents identifying as Swedish and 45% of respondents identifying as Sámi reported exposure to any physical violence; in the SAMINOR 2 study, 10% of male non-Sámi respondents and 19% of male Sámi respondents reported such exposure (Eriksen et al., 2015). There are methodological differences between the current study and the SAMINOR 2 study, especially the wording of the questions about physical and sexual violence. In the current study, examples of physical and sexual violence were given in the questions, but these were not specified in the SAMINOR 2 study. It is possible that some less serious forms of violence, for example, being pushed, were not considered to be violence by respondents in the SAMINOR 2 study, giving rise to a more conservative prevalence estimate of physical violence in the SAMINOR 2 study than in the current study.

The prevalence of emotional violence by all perpetrators was lower in the current study (Swedish female 21%, Sámi female 29%; Swedish male 14%, Sámi male 25%) than SAMINOR 2 (non-Sámi female 26%, Sámi female 39%; non-Sámi male 19%, Sámi male 32%) (Eriksen et al., 2015). The questions about emotional abuse asked in the two studies were almost identical, and hence this does not explain the difference in reported prevalence. In a previous Nordic study using an almost identical item, respondents in Norway also reported a slightly higher prevalence of emotional violence (23%) than respondents in Sweden (19%) (Wijma et al., 2003). Whether these differences should be attributed to methodological or sampling differences, for example, response rate, or to actual differences between the two countries in the prevalence of emotional, physical, or sexual violence is difficult to state with any certainty.

Notably, we found that multiple forms of victimization (reporting two or more types of violence, reporting two or more kinds of perpetrators as well as reporting victimization both as a child and as an adult) were more commonly reported by those identifying as Sámi than Swedish. This indicates that the Sámi population reports more violent experiences overall, and that those affected tend to report a higher burden of victimization. Similarly, Brownridge (2003, 2010) found that Indigenous men and women had overall higher odds of reporting violence, but in particular for reporting the most severe forms of violence. In SAMINOR 2, violence experienced only in adulthood, only in childhood, and in both adulthood and childhood was more common among respondents identifying as Sámi than Swedish (Eriksen et al., 2015). Multiple victimization has repeatedly been found to be associated with worse outcomes for victims than one form of victimization alone (Hamby & Grych, 2013; Nasir et al., 2021; Simmons & Swahnberg, 2021; Simmons et al., 2015; Wiklund et al., 2022).

Interpersonal violence is the result of a complex interplay of factors on the societal, community, relational, and individual levels of the socioecological model. For Indigenous populations, it is particularly important to consider how factors on the societal and community levels, for example, historical losses and discrimination, intersect with factors on the relational and individual levels to increase the risk of interpersonal violence. The mediation analyses conducted in this study revealed that the total effect of identifying as Sámi on reported exposure to violence was mediated by historical losses and discrimination. Hence, identifying as Sámi was positively associated with reporting experiences of historical losses and discrimination, which in turn was positively associated with reporting experiences of violence. These associations explain the greater odds of reporting experiences of violence among those identifying as Sámi than Swedish. Consistent with this, in the fully adjusted logistic regression models, discrimination were found to be strongly associated with increased odds of reporting all forms of interpersonal violence and historical losses was associated with increased odds of reporting all forms of violence, except for violence by a stranger perpetrator. Identifying as Sámi was instead found to be associated with lower odds of reporting any victimization and intimate partner violence in the regression analyses.

This study does not reveal the mechanism by which historical losses and discrimination are associated with different forms of interpersonal violence; rather, it supports the notion that such a link exists and needs to be studied further. Previous studies and theories suggest that lateral violence and intergenerational transmission of violence may explain part of the connection. Lateral violence is said to occur when people of an oppressed group internalize the oppression and adopt the behaviors and practices of their oppressors, including violent behaviors directed at members of their own group (Whyman et al., 2021). Four interconnected factors have been suggested to contribute to the origin of lateral violence among Indigenous populations: (a) Colonization, oppression, and control of Indigenous people, (b) identity conflict/internalization of negative stereotypes, (c) feelings of powerlessness, and (d) loss of land, dismemberment of traditional roles, structures, and knowledge (Sehlin MacNeil, 2024; Whyman et al., 2021). The measures of historical losses and discrimination used in the current study include some of these constructs and were associated with interpersonal violence, supporting the theory of lateral violence.

Violence tends to reoccur across a lifespan, for example, childhood abuse is associated with different forms of violence in adulthood as well as exposure to elder abuse (Hamby & Grych, 2013; Kong & Easton, 2018; Simmons et al., 2014; Wiklund et al., 2022). The knowledge that violence leads to violence may be relevant in explaining the link between historical losses, discrimination, and interpersonal violence found in this study. As previously stated, the colonialism endured by the Sámi population should not only be considered as historical events but affects contemporary Sámi life through the continued experience of historical losses, conflicts over land for reindeer husbandry, fractioned identities, and various forms of discrimination (Axelsson et al., 2019). Colonialism can be considered as structural and cultural violence, which according to Galtung (1990) legitimizing direct forms of interpersonal violence. In addition, historical losses can be considered as a form of indirect violence for the following generations (Hamby et al., 2019). Studies about Indian Residential Schools in Canada have suggested an intergenerational effect wherein the negative effects of the system accumulate, and it has been proposed that the abuse experienced by those forced to attend such schools may have led to normalization of use of violence in relationships (Bombay et al., 2014; Hoffart & Jones, 2018; Kwan, 2015). It has also been suggested that these boarding schools may have fostered children who became adults that were less prepared for parenting through, for example, interrupting the transmission of family values and parenting skills over generations (Evans-Campbell, 2008; Hoffart & Jones, 2018; Kwan, 2015). Such factors could have contributed to high prevalence of childhood abuse in later generations of Indigenous populations.

Older age was associated with lower odds of reporting experiences of all forms of violence, except violence by a family member. This pattern may be attributed to several factors. It is possible that the actual life-course prevalence is lower among older adults, but it is also likely that the recall bias is larger for older adults than for younger adults concerning violence in youth and middle age and this difference should hence be interpreted with caution.

The importance of considering associations between historical losses and discrimination on interpersonal violence should not take focus away from other explanatory factors in the socioecological model, for example, the gendered nature of violence. In this study, reporting historical losses and experiences of discrimination doubled the odds of reporting intimate partner violence, and being female was associated with an eight-fold increase in odds of reporting such violence. A previous study of Indigenous populations in Canada reported gender symmetry for intimate partner violence, that is, men and women reporting similar prevalence of violence (Brownridge et al., 2017). However, in that study a modified version of the Conflict Tactic Scale was used, an instrument that has been criticized for producing gender symmetry (Hamby, 2016). In both this study and the SAMINOR 2 study, items relating to violence were inspired by the NorAQ, which has previously produced gender asymmetry in studies, that is, women reporting higher levels of intimate partner violence than men (Simmons & Swahnberg, 2019). This is a pattern more consistent with other sources of information about intimate partner violence, for example, crime surveys and homicide statistics (Hamby, 2016). Consequently, it is not surprising that women identifying as both Swedish and Sámi were considerably more likely to report intimate partner violence than men in both this study and the SAMINOR 2 study.

In the sample used in this study, 23% of Sámi respondents reported that they had no economic margin, compared to 17% of Swedish respondents. In the final models, this was associated with greater odds of reporting intimate partner violence and any violence victimization. This result is in line with results from Canadian studies indicating that an uneven distribution of risk factors may account for some of the increased odds of reporting exposure to violence among Indigenous populations (Brownridge, 2008; Brownridge et al., 2017). Unexpectedly, social support was only weakly associated with violence by an acquaintance, and alcohol consumption was not associated with reporting violence by any perpetrator. Altogether, our result underlines the importance of using an intersectional approach in studies about interpersonal violence, including, for example, gender and socioeconomic factors as well as historical losses and discrimination in analyses.

### Strengths and Limitations

One strength of this study is that it included both respondents who self-identified as Sámi and as Swedish. Previously, studies have sometimes tried to estimate differences between Indigenous and non-Indigenous populations by comparing studies conducted on the two groups separately (Nasir et al., 2021; Oetzel & Duran, 2004). However, due to the strong effects of methodological choices on reported prevalence of violence (Simmons & Swahnberg, 2019), it is preferable to include both groups in the same study if one wishes to make inferences regarding differences. However, this study also had limitations: one question was used for each of physical, emotional, and sexual violence. Considering that violence is a diverse experience, this may have led to some violent experiences being missed. In addition, no questions about economic violence or neglect were included in the study. The latter would have been particularly interesting given older adults constituted a large proportion of the respondents, and neglect is an important form of elder abuse that has rarely been studied among Indigenous populations. Further, we included neither measure of the frequency of violence nor number of perpetrators, which hampered the consideration of a poly-victimization effect. We used an augmented version of the historical loss scale that has not been validated; in addition, the dichotomization of experiences of historical losses and discrimination are gross simplifications of a complex reality. Also, item nonresponse for historical losses was considerable, at 7.8% among respondents identifying as Swedish and 6.4% among those identifying as Sámi, which can be compared to 0% to 3.4% for the other included items (Table 1). It is possible that this influenced the results.

The response rate was 41%, which might have introduced a nonresponse bias in the study. However, a previous study comparing the prevalence rate of violence reported in different studies in Sweden found that the prevalence was highly dependent on the questionnaire used, but was not related to the response rate (Simmons & Swahnberg, 2019). There were considerable differences in the background characteristics of respondents identifying as Sámi and those identifying as Swedish, and this may have affected the prevalence rates reported. We used population weights to compensate for this, but if a potential nonresponse bias was systematic and not dependent on age, the weighting procedure does not fully compensate for it. Also, because there is no population register including ethnicity in Sweden, it is not possible to know if the nonresponse was similar among those identifying as Swedish and those identifying as Sámi. Altogether, the reported prevalence rates should be interpreted with caution.

In this study, a total population sample from Jokkmokk, an area well known for a large Sámi presence, was used. Based on demographic variables and cultural cohesion, we anticipate that our findings in Jokkmokk can be generalized to Sámi and Swedish populations in other inner parts of northern Sweden and Sápmi. However, we did find differences in prevalence rates between this study and the SAMINOR 2, and it is possible that there is a true difference considering patterns of violence among Sámi and non-Sámi populations in the different parts of Sápmi, including different parts of Sweden and Norway. The Sámi population is heterogeneous and, though the Sámi culture and history are partly communal within the group and across the borders of the two nations, there are also significant differences in terms of living conditions between different groups within the Sámi community. Such differences have not been considered in this study. Altogether, exact prevalence rates should be interpreted with caution. It is especially unfortunate that Sámi were underrepresented among the older respondents, as this may have resulted in an underrepresentation of first- and second-generation victims of colonialism. The mediation models and logistic regression analyses were all adjusted for background characteristics. Hence, the proposed mediating effect of historical losses and discrimination on the effect that identifying as Sámi had on reporting interpersonal violence should be considered an indication that these factors are important to acknowledge in research on interpersonal violence among Indigenous populations. It is, however, important to remember that this was a cross-sectional study, and hence, no inferences about causality should be made.

## Conclusion

This is the first study investigating the prevalence of different forms of interpersonal violence among a sample of respondents identifying as Sámi and Swedish in Sweden. A considerably higher prevalence of violence by acquaintances and strangers was found for respondents identifying as Sámi than those identifying as Swedish. The same pattern was seen for Sámi women concerning violence by a family member; however, in contrast to previous studies of Indigenous populations, intimate partner violence was reported at similar rates by women identifying as Sámi and Swedish. The mediation analyses revealed that the effect of identifying as Sámi on reporting experiences of interpersonal violence was mediated in full by historical losses and discrimination. In addition, gender was found to be an important factor, for example, female respondents had an eight-fold increase in odds of reporting exposure to intimate partner violence while male respondents were more likely to report violence by a stranger perpetrator. Altogether, our findings underline the importance of using an intersectional, life-course, or even intergenerational perspective in research about interpersonal violence, especially among Indigenous populations.

## Supplemental Material

sj-pdf-1-jiv-10.1177_08862605241264544 – Supplemental material for Interpersonal Violence Against Indigenous Sámi and Non-Sámi Populations in Arctic Sweden and the Mediating Effect of Historical Losses and Discrimination Supplemental material, sj-pdf-1-jiv-10.1177_08862605241264544 for Interpersonal Violence Against Indigenous Sámi and Non-Sámi Populations in Arctic Sweden and the Mediating Effect of Historical Losses and Discrimination by Johanna Simmons, Christina Storm Mienna, Maria Josefsson, Per Axelsson and Katarina Nägga in Journal of Interpersonal Violence
