# Supplementary material for: Interpersonal Violence Against Indigenous Sámi and Non-Sámi Populations in Arctic Sweden and the Mediating Effect of Historical Losses and Discrimination
Source: J Interpers Violence. 2024 Sep 10;39(19-20):4135–63. doi: 10.1177/08862605241264544 (PMC11389050; doi:10.1177/08862605241264544)
Supplement: sj-pdf-1-jiv-10.1177_08862605241264544 – Supplemental material for Interpersonal Violence Against Indigenous Sámi and Non-Sámi Populations in Arctic Sweden and the Mediating Effect of Historical Losses and Discrimination [file sj-pdf-1-jiv-10.1177_08862605241264544.pdf]

Table 1. The unweighted (uw) and weighted (w) prevalence of violence among men (n= 628) and women (n=875).

	**Men**							**Women**						
	**Swedish**			**Sámi**				**Swedish**			**Sámi**			
	**n**	**% uw**	**% w**	**n**	**% uw**	**% w**	**p w**	**n**	**% uw**	**% w**	**n**	**% uw**	**% w**	**p w**
**Any violence**	165	33.1	33.5	59	48.0	47.7	**<0.01**	224	36.1	37.0	126	51.4	49.7	**<0.01**
**Age at victimization**							**<0.01**							**<0.01**
No victimization	333	66.9	66.5	64	52.0	52.3		397	63.9	63.0	119	48.6	50.3	
Only childhood	96	19.3	20.1	29	23.6	24.5		73	11.8	12.2	29	11.8	11.9	
Only adulthood	48	9.6	9.4	13	10.6	10.2		98	15.8	16.2	51	20.8	19.5	
Both childhood and adulthood	21	4.2	4.0	17	13.8	13.0		53	8.5	8.7	46	18.8	18.4	
**Type of violence**														
Emotional	72	14.6	14.4	29	24.0	24.7	**<0.01**	125	20.5	21.3	71	29.7	28.8	**0.02**
Physical	134	27.1	27.4	56	45.5	44.7	**<0.01**	139	22.6	23.5	81	33.3	32.8	**<0.01**
Sexual	N/A							115	18.7	19.1	80	33.1	31.7	**<0.01**
**Number of types**							**<0.01**							**<0.01**
No victimization	333	66.9	66.5	64	52.0	52.3		397	63.9	63.0	119	48.6	50.3	
One type	113	22.7	23.3	29	23.6	23.3		112	18.0	18.3	52	21.2	20.3	
Two or more	52	10.4	10.2	30	24.4	24.4		112	18.0	18.7	74	30.2	29.4	
**Kind of Perpetrator**														
Partner	N/A							93	15.0	15.4	35	14.3	13.3	0.443
Family	N/A							54	8.7	9.2	40	16.3	16.4	**<0.01**
Acquaintance	71	14.3	14.8	30	24.6	25.0	**<0.01**	88	14.2	14.7	62	25.3	24.3	**<0.01**
Stranger	72	14.5	14.3	32	26.2	26.3	**<0.01**	53	8.6	8.8	39	15.9	15.8	**<0.01**
**Number of perpetrators**							**<0.01**							**<0.01**
No victimization	333	67.0	66.6	64	52.5	52.8		397	64.2	63.3	119	48.6	50.2	
One perpetrator	146	29.4	29.9	46	37.7	37.6		162	26.2	26.7	83	33.9	32.4	
Two or more perpetrators	18	3.6	3.5	12	9.8	9.6		59	9.5	10.0	43	17.6	17.3	

Note: The weights, i.e. the inverse of the probability of inclusion to the sample, adjusted for age and sex, and were based on population rates in Jokkmokk, 2021 obtained from Statistics Sweden. The differences between groups were calculated using weighted Pearson's chi-square test. Boldface numbers signal differences at the p=0.05 level. N/A= Not applicable, prevalence not reported due to low number of participants.

Table 2. Missing data concerning exposure to violence

	Men				Women			
	**Swedish**		**Sámi**		**Swedish**		**Sámi**	
	**n**	**%**	**n**	**%**	**n**	**%**	**n**	**%**
**Any violence**	7	1.4	0	0.0	8	1.3	1	0.4
**Type of violence**								
Emotional violence	13	2.6	2	1.6	20	3.2	7	2.8
Physical violence	10	2.0	0	0.0	14	2.2	3	1.2
Sexual violence	11	2.2	0	0.0	14	2.2	4	1.6

Note: Respondents with missing values on one or two of the questions about the type of violence (emotional, physical, sexual) were coded as non-exposed to that type of violence when constructing the variables for the kind of perpetrator and repeat victimization. For example, a respondent reporting exposure to emotional violence by an intimate partner perpetrator in adulthood but not answering the questions about physical or sexual violence, was coded as exposed to a) emotional violence, b) intimate partner violence, c) violence only in adulthood, d) one type of violence and e) violence by one kind of perpetrator. This approach was taken to enable inclusion of those respondents in the overall variables constructed for each kind of perpetrator, which include exposure to all types of violence and the variables about repeat victimization, without overestimating the prevalence of violence. Therefore, the missing data on variables about kind of perpetrator and repeat victimization is equivalent to missing data on the overall variable "any violence".
